# Seizure Prediction Model in Acute Tramadol Poisoning; a Derivation and Validation study

**Published:** 2020-05-17

**Authors:** Elham Bazmi, Behnam Behnoush, Saeed Hashemi Nazari, Soheila Khodakarim, Amir Hossein Behnoush, Hamid Soori

**Affiliations:** 1 *Department of Epidemiology, Scho* *ol of Public Health and Safety, Shahid Beheshti University of Medical Sciences, Tehran, Iran. *; 2 *Department of Forensic Medicine, Tehran University of Medical Sciences, Tehran, Iran. *; 3 *Tehran University of Medical Sciences, Tehran, Iran. *; 4 *Safety Promotion and Injury Prevention Research center, Shahid Beheshti University of Medical Sciences, Tehran, Iran. *

**Keywords:** Clinical decision-making, tramadol, poisoning, seizures

## Abstract

**Introduction:**

Seizure is a common complication of tramadol poisoning and predicting it will help clinicians in preventing seizure and better management of patients. This study aimed to develop and validate a prediction model to assess the risk of seizure in acute tramadol poisoning.

**Methods:**

This retrospective observational study was conducted on 909 patients with acute tramadol poisoning in Baharloo Hospital, Tehran, Iran, (2015-2019). Several available demographic, clinical, and para-clinical characteristics were considered as potential predictors of seizure and extracted from clinical records. The data were split into derivation and validation sets (70/30 split) via random sampling. Derivation set was used to develop a multivariable logistic regression model. The model was tested on the validation set and its performance was assessed with receiver operating characteristic (ROC) curve.

**Results:**

The mean (standard deviation (SD)) of patients’ age was 23.75 (7.47) years and 683 (75.1%) of them were male. Seizures occurred in 541 (60%) patients.  Univariate analysis indicated that sex, pulse rate (PR), arterial blood Carbone dioxide pressure (PCO_2_), Glasgow Coma Scale (GCS), blood bicarbonate level, pH, and serum sodium level could predict the chance of seizure in acute tramadol poisoning. The final model in derivation set consisted of sex, PR, GCS, pH, and blood bicarbonate level. The model showed good accuracy on the validation set with an area under the ROC curve of 0.77 (95% CI: 0.67–0.87).

**Conclusion:**

Representation of this model as a decision tree could help clinicians to identify high-risk patients with tramadol poisoning-induced seizure and in decision-making at triage of emergency departments in hospitals.

## Introduction

Tramadol is a synthetic opioid analgesic used for alleviation of moderate to severe pain. This drug weakly binds to µ opioid receptors and inhibits the reuptake of monoamines such as serotonin and norepinephrine in the central nervous system (1, 2). 

In recent years, easy and wide availability, excessive prescription, and euphoria effects of this drug have caused a rapid increase in tramadol consumption and poisoning in Iran (3-5). 

 S*eizure* is a common, serious neurological side effect of tramadol consumption in various doses, which is associated with several complications such as lactic acidosis and rhabdomyolysis (6). Although, previous investigations indicated that tramadol-induced seizure occurrs due to inhibition of gamma amino butyric acid (GABA) receptors and serotonin toxicity, the exact mechanism of tramadol induced seizure is still unknown (7-9).

Several studies have shown associations between seizure and related factors such as opioid dependency, age, sex, consumption dose, blood concentration, and delayed hospital admission, but their effects are still under debate and other related factors have remained unclear (5, 10-12). 

Having a prediction model that discriminates patients who will develop seizure, can help clinicians in quickly detecting high-risk patients and immediately taking suitable action. 

Several investigations have been done to develop prediction models of several types of seizure by using effective factors and various analytic methods such as frequency–based methods, statistical analysis of EEG signals, least absolute shrinkage and selection operator (LASSO) regression, non-linear dynamics (chaos), logistic regression and machine learning models like support vector machine (SVM) (13-16). 

In spite of various reports of research, to date, no prediction model is available for tramadol induced seizure. A prediction model should consist of variables that are readily available in clinic and needs to be parsimonious (17). Also, in previously suggested prediction models, there is not a suitable trade-off between simplicity and accuracy of model, and they are not widely applicable in the emergency department due to the complexity of their utilization as a clinical tool.

This study aimed to develop and validate a prediction model to assess the risk of seizure in acute tramadol poisoning by using demographic, clinical and para-clinical factors of patients admitted to the emergency department of a hospital in a regression model and developing a decision tree based on the final model as a clinical decision instrument. 

## Methods

***Study Design and Setting***

This retrospective, single center, cohort study of routinely collected clinical data was performed on acute tramadol intoxicated patients who had referred to the emergency department (ED) of Baharloo Hospital (a poisoning referral center), Tehran, Iran, in the 5-year period between September 2015 and November 2019. Several available demographic, clinical, and laboratory characteristics were considered as potential predictors of seizure and extracted from clinical records. The data were split into derivation and validation sets (70/30 split) using random sampling. Derivation set was used to develop a multivariable logistic regression model. The model was tested on the validation set and its performance was assessed via receiver operating characteristic (ROC) curve. This study was approved by ethical committee of Shahid Beheshti University of Medical Sciences (IR.SBMU.PHNS.REC.1398.110). All individual information was kept confidential and data analysis was done anonymously. In this research, we followed the reporting guideline from the TRIPOD (Transparent Reporting of a multivariable prediction model for Individual Prognosis or Diagnosis) statement (18).

***Participants***

All patients with a history of tramadol overdose, confirmed by laboratory test results, were included in this research (909 patients). We excluded patients aged less than 15 years, as well as those with history of renal, hepatic, cardiovascular and respiratory disorders, epilepsy, co-ingestion of other drugs, recent seizure history, pregnancy and missing data in clinical records.

***Data gathering***

The data were extracted from clinical records and hospital’s Electronic System, which were registered based on the examination of patients at the time of admission to ED performed by two trained researchers. Extracted data included demographic, clinical, and laboratory ones.

 As the predictors for development of model, we included routinely available information at ED settings. Demographic variables consisted of age, sex, ingested dose, history of opioid addiction, time elapsed from consumption, and manner of poisoning; clinical variables consisted of systolic blood pressure, diastolic blood pressure, Glasgow Coma Scale (GCS), pulse rate (PR), respiratory rate (RR), and laboratory variables included arterial blood oxygen pressure (PO_2_), arterial blood Carbone dioxide pressure (PCO_2_), blood oxygen saturation levels (O_2_Sat),  blood bicarbonate level,  platelet count, hemoglobin level (Hb), white blood cell count (WBC), blood sugar, and serum sodium and potassium levels. 

***Reference standard***

In this study, the gold standard was occurrence of seizure, defined as an episode of neurologic dysfunction caused by abnormal neuronal activity that results in a sudden change in behavior, sensory perception, or motor activity (19). Tramadol-induced seizures, as outcome of this research, are frequently reported to be generalized, tonic-clonic in nature, and without auras or focal symptoms, and occur during the first 24 hours after admission to ED and are diagnosed via clinical observations and confirmed using electroencephalogram (EEG) in suspected patients.

***Statistical analysis***

Information of 909 patients with acute tramadol poisoning who were admitted to ED was evaluated in this study. The analyses were performed using Stata software version 16.1 and R software version 3.6.2.

Data were split into derivation (70%) and validation sets (30%) using random sampling. Data in the derivation set were applied to develop prediction models and data of validation set were used to evaluate the model’s performance and compare predicted probability with actual patient outcomes.

In descriptive statistics, the baseline characteristics and prevalence of seizure were analyzed in both derivation and validation datasets to assure similarity. 

In derivation set, predictor variables were identified via univariate logistic regression analysis, performed on all variables to assess their ability to predict seizure. 

The prediction model was developed by using stepwise logistic regression on the derivation set. We trained several models to choose potential contributing predictors, which were included in the final prognostic model (p value<0.05 was considered as statistically significant in stepwise selection). 

The performance of the final model, including its discrimination and calibration, was evaluated in both derivation and validation sets. The discrimination of the model was measured via k-fold cross validation (k=10) method using the area under the curve (AUC) in the receiver operating characteristic (ROC) analysis.

The calibration of the model was assessed via Hosmer-Lemeshow statistic and plotting the calibration curve with “caret” package in R and creating 10 bins for predicted probabilities of seizure and choosing the bin midpoints for observed seizure rates. 

Also, prediction performance was evaluated using confusion matrix results such as sensitivity, specificity, positive predictive values (PPV), negative predictive values (NPV) of the final model in the validation set.

We developed a decision tree plot to represent choices and model results to the risk of seizure in tramadol poisoning. The nodes in the graph represent an event (Seizure=Yes and Seizure=No) and the edges of the graph indicate the decision rules. 

## Results

***Baseline characteristic of patients***

**Figure 1:**
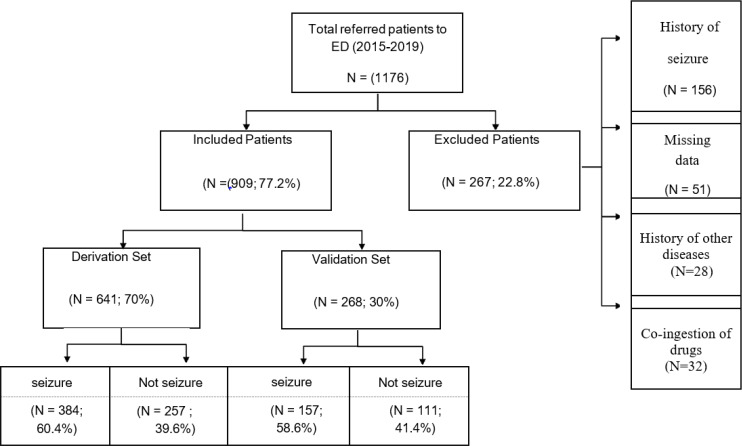
Flow of all patients referred to the emergency department from September 2015 to November 2019 in seizure prediction study

In this study, 1176 patients with acute tramadol poisoning were identified. Among them, 267 patients were excluded and 909 patients were enrolled in our investigation. Figure 1 shows the flow of the total number of patients referred to the ED in our dataset. 

The mean (standard deviation (SD)) age of the patients was 23.75 (7.47) years, their age range was 16-65 years and 683 (75.1%) of them were male. The most common cause of poisoning was suicide, which occurred in 644 (71%) patients and 427 (47%) subjects had a history of addiction to opioids. Seizure occurred in 541 (59.8%) of the patients with acute tramadol toxicity.

The time interval between tramadol ingestion and hospital admission was 4.95 ± 4.1 hours and the mean (SD) of the last dose of tramadol consumption was 1770 ± 918.8 mg. Demographic, clinical, and laboratory characteristics of patients in derivation and validation sets are shown in table1. The baseline characteristics and proportion of patients who experienced seizure on the derivation and validation datasets were similar. 

***Development of prediction model***

Based on univariate analysis, being male (OR=2.39; 95% CI:1.47-3.87), increase in pulse rate (OR=1.33; 95% CI:1.11-1.45) and arterial blood carbon dioxide pressure (OR=1.16;95%CI:.1.1-1.22) and decrease in GCS (OR=0.88; 95%CI:0.78-0.99), blood bicarbonate level (OR=0.9; 95%CI: 0.832-.937), pH (OR=0.26; 95%CI: 0.20-0.31), and sodium level (OR=0.95; 95%CI:0.9-0.99) significantly increased the chance of seizure in acute tramadol poisoning.

The final model obtained by multivariable logistic regression analysis with corresponding adjusted odds ratio and 95% CI in derivation set is shown in table 2. In this model, male sex, high pulse rate, low pH, low blood bicarbonate level, and low GCS would significantly increase the probability of tramadol-induced seizure.

***Screening Performance of prediction model***

**Figure 2:**
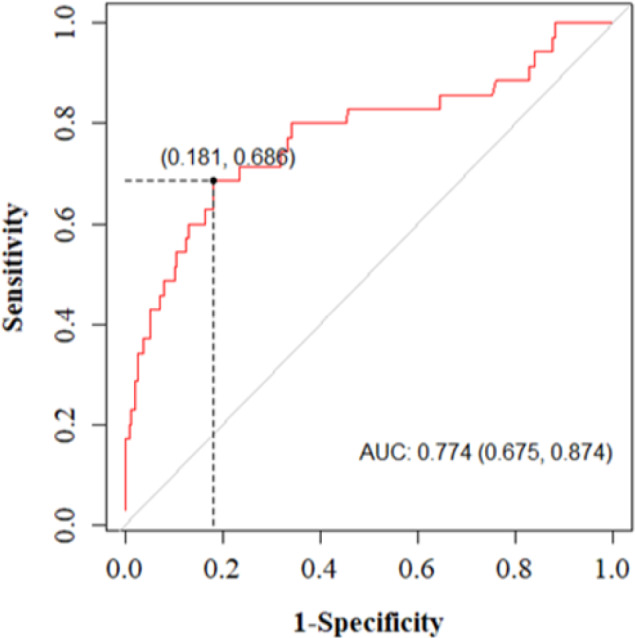
Receiver operating characteristic (ROC) curve of the seizure prediction model in validation set

In evaluating the performance of the model in derivation and validation sets, area under the ROC curve (AUC) of the final model were 0.791 (95% CI: 0.713-0.910) and 0.774 (95% CI: 0.675-0.874), respectively, which indicated good discriminatory power. The curves are shown in figure 2.

The optimal threshold cut-off value was 0.6, which was determined by the highest Youden Index value. In the final model, this maximized sensitivity (0.80; 95% CI: 0.745-0.890) and specificity (0.60; 95% CI: 0.588-0.68). Positive predictive value (PPV) was 0.71 (95% CI: 0.63-0.76) and negative predictive value (NPV) was 0.64 (95% CI: 0.580-0.71) in our data. The performance characteristics of the model in derivation and validation sets are presented in table 3.

**Figure 3:**
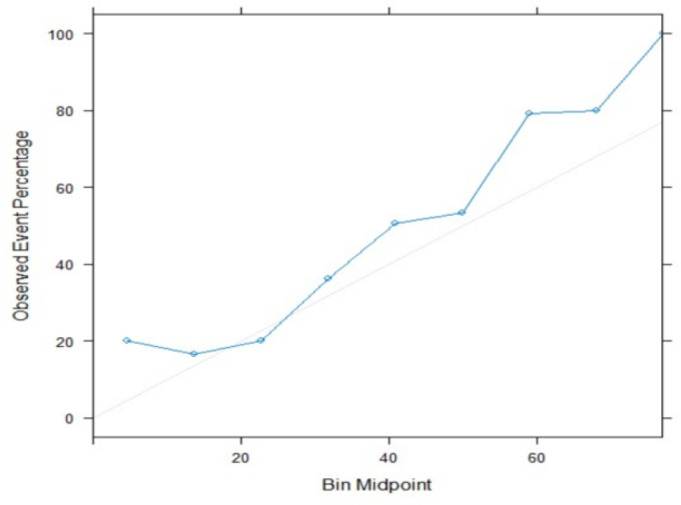
Calibration plot of the seizure prediction model in validation set

Figure 3 shows good agreement between the predicted and observed cases of seizure in the calibration curve of our prediction model in validation set.

***Decision tree***

**Figure 4:**
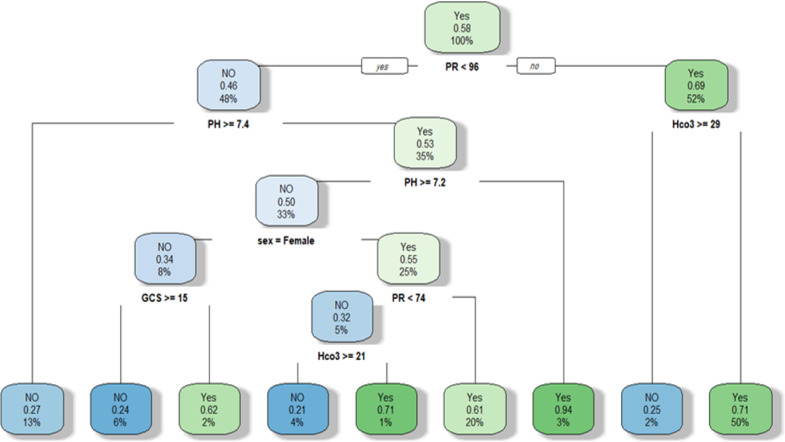
The decision tree plot to assess the risk of seizure in acute tramadol poisoning

We suggested the model with 5 potential predictors including sex, PR, GCS, blood bicarbonate level and pH and presented it as a decision tree in figure 4.

At the top of the plot, the overall probability of seizure was observed (0.58). Therefore, the node asks whether the PR of patients is lower than 96. If yes, then it goes down to the root's left child node. 48% of patients had PR<96 with a seizure probability of 0.46. In the second node, if the PH is higher than 7.4, the chance of seizure occurrence is 0.27.

## Discussion

In this research, we developed a prognostic model to identify patients at high risk for seizure among those with acute tramadol poisoning by using routinely available demographic, clinical and laboratory predictors and validated the model by applying it on another dataset.

In this study, the rate of seizure following tramadol poisoning (60%) was similar to previous reports in Iran, which reported that seizure occurred in 15% to 65% of tramadol poisoned patients. Different seizure rate ranges in other poisoning centers are due to differences in study methods, sample sizes and dose and pattern of tramadol consumption (20-24). 

The wide range of the last dose of ingested tramadol (100 to 3000 mg) in this investigation indicated seizures due to tramadol toxicity were dose-independent. Therefore, reported dose of consumption could not predict seizure. This finding is consistent with the results of other investigations (22, 25, 26). Previously, another research had reported that although higher doses of tramadol correlated with higher blood concentration, it was not associated with seizure (21, 25). On the other hand, some studies reported dose-dependent characteristics of seizure in tramadol overdose (8, 23). The reason for this inconsistency is drug dependency and individuals’ tolerance. Also, various racemic formulations of tramadol in Iran cause different pharmacokinetics and pharmacodynamic characteristics (27). 

Difference between the minimum dose of tramadol consumed in our study and other investigations is due to purity and concentration of active ingredients' formulation of tramadol tablets, ambiguity about quantity of ingested tables, and their dose in patients with seizures in these studies (22).

In our research, the mean time interval between drug ingestion and seizure was 5 hours (range: 30 minutes to 24 hours) and it usually occurred in the first 24 hours after consumption, which is consistent with other investigations (6, 21, 22, 25).

Most of the patients with acute tramadol toxicity were male with the mean age of 23.75±7.46 years, which is in line with other reports (26, 28, 29). Our findings could be explained by increasing drug abuse among young men trying to distance themselves from social and financial problems by using opioid drugs such as tramadol.

Predictive variables provide the full multivariate model with high discriminative ability, which is the result of repeated clinical practices in emergency department of hospitals. Previous studies indicated that a model with multiple variables, such as our full model, which is based on demographic, clinical and laboratory characteristics, has better performance in prediction of seizure compared to models with only one of them (such as EEG) (6, 29).

In our study, patients with lower blood bicarbonate level (<17.1) and lower pH (<7.27) were at high risk for seizure within the initial 24 hours after admission to emergency departments. The results of other studies showed that pH, lactate and other blood gases could predict occurrence and recurrence of seizure, which is in line with our findings. Also, other research reported that, serum laboratory testing and arterial blood gas analysis might be helpful for differentiating between generalized seizure and syncope in patients who experienced a transient loss of consciousness and were referred to the emergency department (30-34).

Pulse rate was an important factor for tramadol poisoning-induced seizure prediction in our research, which is similar to other studies. The results of these investigations proposed seizures prediction algorithms based on heart rate variation and ECG changes. Although, the recording of ECG was much easier and faster than electroencephalogram (EEG), ECG and PR had less value than EEG (35, 36). 

Our findings showed that sodium blood level significantly correlated with the occurrence of seizures, which was confirmed in other investigations. Moreover, the clinical manifestations of hyponatremia were associated with CNS dysfunctions, a rapid decrease in serum sodium level in routine laboratory findings could cause neuronal activity depression and cerebral edema with neurologic symptoms such as EEG changes and seizures (37, 38).

The results of our logistic regression analysis showed that simple and easy blood tests could be a valuable help for clinicians in predicting tramadol poisoning-induced seizure by determining electrolyte levels and blood gas pressure.

The analysis of receiver operation characteristics (ROC) curve of sex, GCS, pH, blood bicarbonate level and pulse rate as predictors of seizure occurrence showed a cut-off value of 0.66, 0.63, 0.63, 0.67, and 0.67, respectively that were able to predict 0.77 of cases who would develop seizures.

The main strength of our prediction model is the size of the data set used for its development. This is among the largest data sets used to develop a seizure prediction model in tramadol toxicity. Also, due to simplicity and good calibration and discrimination of our model, we could have presented this model as a nomogram to calculate the probability of seizure occurrence at the time of patients’ presentation to ED. 

**Table 1:** Demographic, clinical and laboratory characteristics of patients in derivation and validation sets of seizure prediction model in acute tramadol poisoning

**Characteristics**	**Derivation set (n=641)**	**Validation set (n=268)**
**Age (year)**		
Mean ± SD	23.3 ± 7.2	24.6 ± 7.8
**Sex**		
Male	485(75.7)	198(74)
Female	156(24.3)	70(26)
**Last dose of ingestion (mg)**		
Mean ± SD	1736 ± 170	1783 ± 209
**Ingestion to admission (hour)**		
Mean ± SD	5 ± 4.1	4.7± 3.3
**Addiction History**		
Yes	304 (47.4)	123(45.9)
No	337 (52.6)	145(54.1)
**Cause of poisoning**		
Suicide	453 (70.7)	191 (71.3)
Accidental	20 (3.1)	11 (4.1)
Overdose	142 (22.2)	54 (20.1)
Unknown	24 (3.7)	11 (4.1)
**Vital signs**		
Systolic blood pressure (mmHg)	121.8 (16.2)	122.1 (15.4)
Diastolic blood pressure (mmHg)	75.1 (11.4)	74.5 (10.9)
Respiratory rate (breath/minute)	75.1 (11.2)	76.3 (13.4)
Pulse rate (pulse/minute)	98.2 (20.5)	98.9 (19.6)
Glasgow Coma Scale	14.1 (1.9)	14.1 (1.8)
**Laboratory**		
White blood cell (10^3^/mm)	10.5(3.6)	10.7 (3.5)
Hemoglobin (g/dL)	14.6 (2.4)	14.5 (2.3)
Platelet (10^3^/μL)	239.7 (72.3)	245.6 (81.9)
pH	7.3 (.12)	7.2 (.09)
PO_2_ (mmHg)	81.5 (23.8)	82.9 (19.9)
PCO_2_ (mmHg)	43.9 (10)	44.2 (10.7)
Blood Bicarbonate level (mmol/l)	22 (3.8)	21.9 (4)
O_2_ saturation (%)	0.92 (.06)	0.93 (.07)
Blood Sugar (mg/dl)	110.3 (77.1)	109.3 (38.6)
Sodium level (meq/l)	140.7 (4.1)	141.3 (3.7)
Potassium level (meq/l)	3.9 (.3)	3.8 (.2)

Data are presented as mean ± standard deviation (SD) or frequency (%).

**Table 2:** Final variables included in the prediction model in multivariate logistic regression model in derivation set

**Variables**	**RC**	**SE**	**Wald test**	**p value**	**OR (%95 CI)**
Pulse Rate	.015	.004	3.38	.0007	1.015(1.01-1.02)
Glasgow Coma Scale	-.158	.056	-2.8	.0051	.871(.78-.973)
pH	-2.334	.892	-2.61	.0091.006-	.106(.018-.618)
Blood Bicarbonate level	-.106	.024	-4.42	<0.0001	.905(.863-.949)
Sex	.845	.201	4.19	<0.0001	2.348(1.58-3.5)
Intercept	26.63	7.54	3.53	0.0004	1222299928.3

RC: regression coefficient; OR: Odds ratio; SE: standard error; CI: Confidence Interval.

**Table 3:** The Screening performance characteristics of the model in derivation and validation sets

**Characteristics**	**Derivation set**	**Validation set**
Cut-point	0.60 (0.55-0.68)	0.60 (0.55-0.68)
Sensitivity	0.85 (0.81-0.88)	0.80 (0.78-0.88)
Specificity	0.40 (0.36-0.48)	0.60 (0.56-0.67)
Positive predictive values	0.73 (0.69-0.74)	0.71 (0.63-0.76)
Negative predictive values	0.67 (0.61-0.70)	0.64 (0.58-0.71)
Positive likelihood ratio	0.63 (0.60-0.67)	0.40 (0.36-0.43)
Negative likelihood ratio	0.41 (0.35-0.43)	0.35 (0.31-0.39)
Area under curve	0.79 (0.71-0.76)	0.77 (0.70-0.8)
Accuracy	0.73 (0.72-0.78)	0.75 (0.70-0.79)
Hosmer- Lemeshow K^2 ^(p value)	9.4 (0.31)	2.7 (0.95)

All data are presented with 95% confidence interval (CI).

Example: At the top of the plot, the overall probability of seizure is shown (0.58). Therefore, the node asks whether the pulse rate (PR) of patients is lower than 96. If yes, then it goes down to the root's left child node. 48% of patients had PR<96 with a seizure probability of 0.46. In the second node, if the PH is higher than 7.4, the chance of seizure occurrence is 0.27. Yes and no in the nodes indicate the prediction of seizure occurrence.

## Conclusion:

In this investigation, a validated model was developed to predict seizure in acute tramadol poisoning cases based on readily available demographic, clinical and laboratory information. Presentation of this model as a simple and easy to use nomogram could help clinicians to identify high risk patients for tramadol induced seizure and facilitate decision-making at triage of emergency departments in hospitals.

## Limitations

There were several limitations in this study. Firstly, we excluded samples with missing information, which could have affected our data analysis. Yet, missing data was negligible (less than 6%). Secondly, our final prediction model was based on data from a single center. Therefore, development of an algorithm using multicenter data to confirm this model seems necessary.
